# Supplementary figures and images for: Spatiotemporal distribution of cutaneous leishmaniasis in Sri Lanka and future case burden estimates
Source: PLoS Negl Trop Dis. 2021 Apr 23;15(4):e0009346. doi: 10.1371/journal.pntd.0009346 (PMC8099137; doi:10.1371/journal.pntd.0009346)

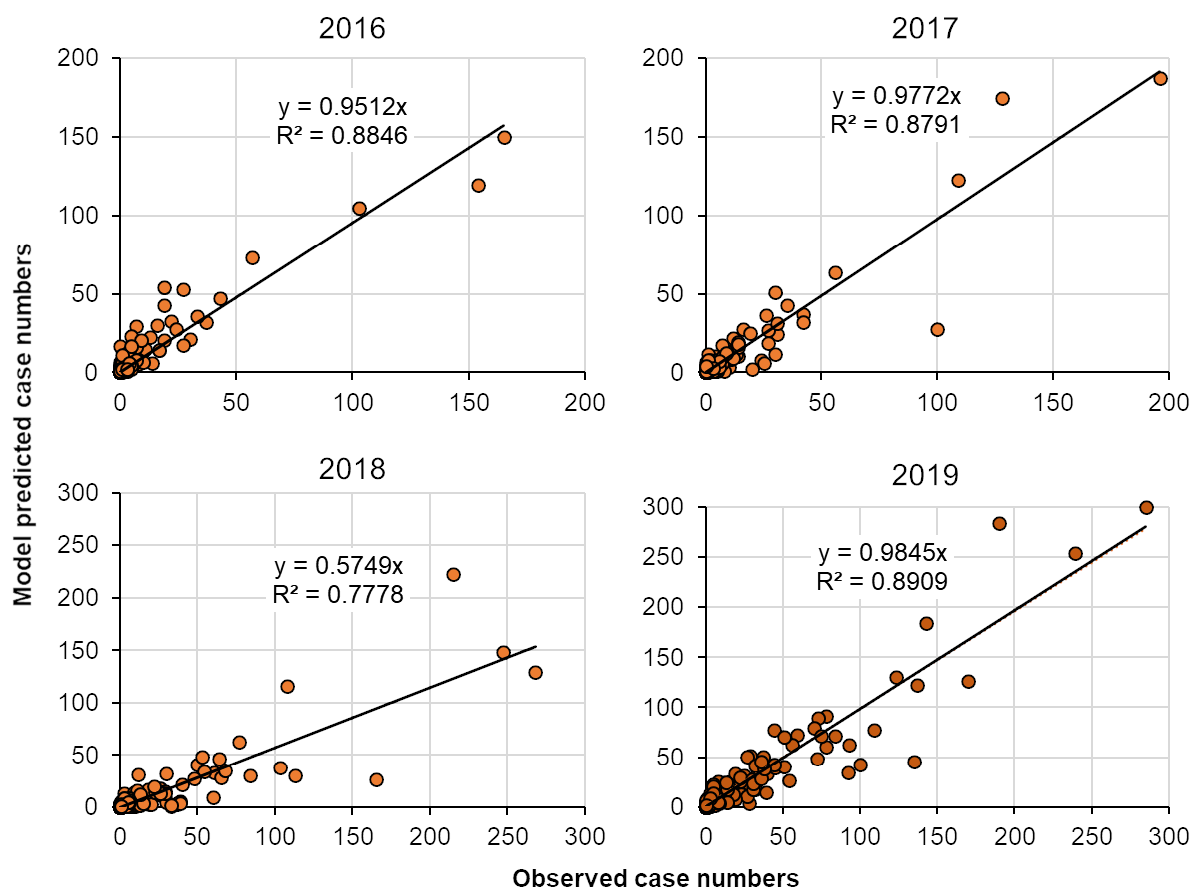

Supplement: S1 Fig — (TIF) [file pntd.0009346.s001.tif]
