# Supplementary material for: Spatiotemporal distribution of cutaneous leishmaniasis in Sri Lanka and future case burden estimates
Source: PLoS Negl Trop Dis. 2021 Apr 23;15(4):e0009346. doi: 10.1371/journal.pntd.0009346 (PMC8099137; doi:10.1371/journal.pntd.0009346)

PLOS Neglected Tropical Diseases

Spatiotemporal transmission of cutaneous leishmaniasis in Sri Lanka and future case burden estimates

--Manuscript Draft--

**Manuscript Number:**	PNTD-D-20-02228
**Full Title:**	Spatiotemporal transmission of cutaneous leishmaniasis in Sri Lanka and future case burden estimates
**Short Title:**	Cutaneous leishmaniasis transmission trends in Sri Lanka and future disease predictions
**Article Type:**	Research Article
**Keywords:**	Leishmania donovani; epidemiology; disease burden; local and inter-regional transmission; protozoa; cutaneous leishmaniasis; skin lesions; infectious diseases
**Abstract:**	Background<br>Leishmaniasis is a neglected tropical vector-borne disease, which is on the rise in Sri Lanka, in contrast to the declining trend reported in other South Asian countries. Spatiotemporal and risk factor analyses are useful for understanding transmission dynamics and predicting future disease distribution to facilitate effective infection control.<br>Methods<br>We collected nationwide clinical cutaneous leishmaniasis and climatic data from 2001 to 2019. Hierarchical clustering and spatiotemporal cross-correlation analysis were used to measure the region-wide and local (between neighboring districts) synchrony of transmission. A mixed spatiotemporal regression-auto regression model was built to study the effects of climatic, neighboring-district dispersal, and infection carryover variables on leishmaniasis dynamics. Same model without climatic variables was used to predict the future distribution of leishmaniasis cases in Sri Lanka.<br>Results<br>There were three phases identified: low-transmission phase (2001–2010), parasite population buildup phase (2011–2017), and outbreak phase (2018–2019). Spatially, the districts were divided into three groups based on similarity in temporal dynamics. The global mean correlation among district incidence dynamics was 0.30 (95% CI 0.25–0.35), and the localized mean correlation between neighboring districts was 0.58 (95% CI 0.42–0.73). Risk analysis for the seven districts with the highest incidence rates indicated that precipitation, neighboring-district effect, and infection carryover effect exhibited significant correlation with district-level incidence dynamics. Model-predicted incidence dynamics and case distribution matched well with observed results, except for the outbreak in 2018. The model-predicted 2020 case number is about 5,400 cases, with intensified transmission and expansion of high-transmission area. The predicted case number will be 9115 in 2022 and 19212 in 2025.<br>Conclusions<br>Leishmaniasis cases in Sri Lanka have gradually increased from 2001 to 2017, with a drastic upsurge thereafter. Transmission was strongly linked to precipitation, inter-district dispersal, and high burden of localized infections. Targeted interventions are urgently needed to arrest an uncontrollable disease spread.
**Additional Information:**	
**Question**	**Response**
**Financial Disclosure**<br><br>Enter a financial disclosure statement that describes the sources of funding for the work included in this submission. Review the submission guidelines for detailed requirements. View published research articles from *PLOS NTDs* for specific examples.	Research reported in this publication was supported by the National Institute of Allergy and Infectious Diseases, National Institutes of Health under award no. U01AI136033 to N.D.K. and G.Z. The authors are solely responsible for the content and it does not necessarily represent the official views of the National Institutes of Health. The funders had no role in study design, data collection and analysis, decision to publish or preparation of the manuscript.

This statement is required for submission and **will appear in the published article** if the submission is accepted. Please make sure it is accurate.<br><br>**Unfunded studies**<br><br>Enter: *The author(s) received no specific funding for this work.*<br><br>**Funded studies**<br><br>Enter a statement with the following details:<br>• Initials of the authors who received each award<br>• Grant numbers awarded to each author<br>• The full name of each funder<br>• URL of each funder website<br>• Did the sponsors or funders play any role in the study design, data collection and analysis, decision to publish, or preparation of the manuscript?<br>• **NO** - Include this sentence at the end of your statement: *The funders had no role in study design, data collection and analysis, decision to publish, or preparation of the manuscript.*<br>• **YES** - Specify the role(s) played.<br><br>* typeset	
**Competing Interests**<br><br>Use the instructions below to enter a competing interest statement for this submission. On behalf of all authors, disclose any competing interests that could be perceived to bias this work—acknowledging all financial support and any other relevant financial or non-financial competing interests.	The authors have declared that no competing interests exist.

This statement **will appear in the published article** if the submission is accepted. Please make sure it is accurate. View published research articles from *PLOS NTDs* for specific examples.<br><br>**NO authors have competing interests**<br><br>Enter: *The authors have declared that no competing interests exist.*<br><br>**Authors with competing interests**<br><br>Enter competing interest details beginning with this statement:<br><br>*I have read the journal's policy and the authors of this manuscript have the following competing interests: [insert competing interests here]*<br><br>* typeset	
**Data Availability**<br><br>Authors are required to make all data underlying the findings described fully available, without restriction, and from the time of publication. PLOS allows rare exceptions to address legal and ethical concerns. See the PLOS Data Policy and FAQ for detailed information.<br><br>A Data Availability Statement describing where the data can be found is required at submission. Your answers to this question constitute the Data Availability Statement and **will be published in the article**, if	Yes - all data are fully available without restriction

accepted.<br><br>**Important:** Stating 'data available on request from the author' is not sufficient. If your data are only available upon request, select 'No' for the first question and explain your exceptional situation in the text box.<br><br>Do the authors confirm that all data underlying the findings described in their manuscript are fully available without restriction?	
**Describe where the data may be found in full sentences. If you are copying our sample text, replace any instances of XXX with the appropriate details.**<br><br>• If the data are **held or will be held in a public repository**, include URLs, accession numbers or DOIs. If this information will only be available after acceptance, indicate this by ticking the box below. For example: *All XXX files are available from the XXX database (accession number(s) XXX, XXX.).*<br>• If the data are all contained **within the manuscript and/or Supporting Information files**, enter the following: *All relevant data are within the manuscript and its Supporting Information files.*<br>• If neither of these applies but you are able to provide **details of access elsewhere**, with or without limitations, please do so. For example:<br><br>*Data cannot be shared publicly because of [XXX]. Data are available from the XXX Institutional Data Access / Ethics Committee (contact via XXX) for researchers who meet the criteria for access to confidential data.*	Data is available with authors.

The data underlying the results presented in the study are available from (include the name of the third party and contact information or URL).<br>• This text is appropriate if the data are owned by a third party and authors do not have permission to share the data.<br><br>* typeset	
Additional data availability information:	Tick here if the URLs/accession numbers/DOIs will be available only after acceptance of the manuscript for publication so that we can ensure their inclusion before publication.

Powered by Editorial Manager® and ProduXion Manager® from Aries Systems Corporation

Manuscript

Click here to access/download;Manuscript;MS Text_Final_Format.docx

Research article

Full title:

Spatiotemporal transmission of cutaneous leishmaniasis in Sri Lanka and future case burden estimates

Short title:

Leishmaniasis transmission trends in Sri Lanka and future disease predictions

Nadira D. Karunaweera[1+*], Sanath Senanayake[1], Samitha Ginige[2], Hermali Silva[1], Nuwani Manamperi[3], Nilakshi Samaranayake[1], Rajika Dewasurendra[1], Panduka Karunanayake[1], Deepa Gamage[2], Nissanka de Silva[4], Upul Senerath[1], and Guofa Zhou[5+]

Author affiliations:

[1] Faculty of Medicine, University of Colombo, Sri Lanka

[2] Ministry of Health, Sri Lanka

[3] Faculty of Medicine, University of Kelaniya, Sri Lanka

[4] Faculty of Science, University of Sri Jayawardanapura, Sri Lanka

[5] University of California Irvine, Irvine, USA

[+] These authors were co-principal investigators.

* Corresponding author

Mailing address of corresponding author:

Nadira D. Karunaweera, Department of Parasitology, Faculty of Medicine, 25 Kynsey Road, Colombo 0008, Sri Lanka. Email: nadira@parasit.cmb.ac.lk Telephone: +94 11 2699284

Abstract

Background

Leishmaniasis is a neglected tropical vector-borne disease, which is on the rise in Sri Lanka, in contrast to the declining trend reported in other South Asian countries. Spatiotemporal and risk factor analyses are useful for understanding transmission dynamics and predicting future disease distribution to facilitate effective infection control.

Methods

We collected nationwide clinical cutaneous leishmaniasis and climatic data from 2001 to 2019. Hierarchical clustering and spatiotemporal cross-correlation analysis were used to measure the region-wide and local (between neighboring districts) synchrony of transmission. A mixed spatiotemporal regression-auto regression model was built to study the effects of climatic, neighboring-district dispersal, and infection carryover variables on leishmaniasis dynamics. Same model without climatic variables was used to predict the future distribution of leishmaniasis cases in Sri Lanka.

Results

There were three phases identified: low-transmission phase (2001–2010), parasite population buildup phase (2011–2017), and outbreak phase (2018–2019). Spatially, the districts were divided into three groups based on similarity in temporal dynamics. The global mean correlation among district incidence dynamics was 0.30 (95% CI 0.25–0.35), and the localized mean correlation between neighboring districts was 0.58 (95% CI 0.42–0.73). Risk analysis for the seven districts with the highest incidence rates indicated that precipitation, neighboring-district effect, and infection carryover effect exhibited significant correlation with district-level incidence dynamics. Model-predicted incidence dynamics and case distribution matched well with observed results, except for the outbreak in 2018. The model-predicted 2020 case number is

about 5,400 cases, with intensified transmission and expansion of high-transmission area. The predicted case number will be 9115 in 2022 and 19212 in 2025.

Conclusions

Leishmaniasis cases in Sri Lanka have gradually increased from 2001 to 2017, with a drastic upsurge thereafter. Transmission was strongly linked to precipitation, inter-district dispersal, and high burden of localized infections. Targeted interventions are urgently needed to arrest an uncontrollable disease spread.

Keywords

Leishmania donovani, epidemiology, disease burden, local and interregional transmission, protozoa, cutaneous leishmaniasis, skin lesions, infectious diseases.

Author summary

Leishmaniasis is on the rise in Sri Lanka in contrast to the declining trend in rest of South Asia. Spatiotemporal analysis and disease risk factors are useful for understanding transmission mechanisms and predicting future disease distribution to facilitate control. In this study we analyzed data on cutaneous leishmaniasis cases from Sri Lanka from 2001 to 2019. We asked three important questions regarding the driving forces behind the intensified leishmaniasis transmission: 1) Are the transmission dynamics in different areas synchronized? 2) What is the role of neighboring-area dispersal in shaping transmission dynamics? 3) How important is climatic variability in transmission dynamics? We used a multi-step approach to answer these questions. In addition to cross-correlation analysis, we built a mixed spatiotemporal regression-autoregression model to analyze risk factors, which is unique in leishmaniasis research because the simplified model was also useful for predicting future disease distribution. We found that the incidence dynamics in different districts could be divided into three synchronized groups based on similarity. Risk factor analysis indicated that precipitation, neighboring-district dispersal, and local infection carryover played important roles in shaping transmission dynamics. The spatiotemporal model predicted intensifying transmission with increasing case numbers, and expansion of high-transmission areas. Targeted interventions are urgently needed to stem the outbreak.

Introduction

Leishmaniases are neglected tropical diseases caused by *Leishmania* parasites and transmitted through the bites o[illegible]ector sand flies [1]. There are three main types of leishmaniasis: visceral (VL), the most serious form of the disease; cutaneous (CL), the most common; and mucocutaneous (MCL), the most disabling form of the diseas[illegible]inety two countrie[illegible] territories remain endemic for leishmaniasis, with more than one billion people living at risk of acquiring the disease [1]. There are about 30,000 new cases of VL and >1 million new cases of CL that occur annually [1]. Although nearly 100 countries are endemic to leishmaniases, major transmissions are concentrated within a few. In 2018, over 90% of global VL cases were reported from seven countries: Brazil, Ethiopia, India, Kenya, Somalia, South Sudan and Suda[illegible] Over 90% of MCL cases occurred in Bolivia, Brazil, Ethiopia and Peru, and 10 countries reported more than 5000 CL cases [1, 2].

Three South Asian countries, India, Nepal, and Bangladesh, accounted for about half the global burden of VL [2]. Encouraged by the recent progress achieved in leishmaniasis control, these countries made a commitment to eliminate VL in the region by 2015 with the deadline extended thereafter [3, 4]. The elimination target was set at less than one case per 10,000 people per year, an incidence rate considered to be no longer of public health concern [3, 4]. However, Sri Lanka, India’s neighbor, has reported a substantial surge in clinical leishmaniasis cases in the past 20 years [5]. The vast majority of leishmaniasis clinical cases in Sri Lanka are CL caused by *Leishmania donovani*, the same parasite that causes VL elsewhere, including in India [2, 6]. The probable vector of the Sri Lankan leishmaniasis parasite is the *Phlebotomus argentipes glaucus* sand fly, which demonstrates zoophilic behavior and differs from the *Phlebotomus* species of sand flies found in southern India near the India – Sri Lanka strait [7, 8]. Previous studies found that leishmaniasis epidemics in Sri Lanka had two transmission hotspots, one on the south coast and another in the north central region of the country, with a possible biannual

seasonal variation [5, 9]. Clinical leishmaniasis in Sri Lanka also showed a shift in sex and age distribution, from predominantly males aged 21–40 in early 2000 to a more balanced sex ratio and a 50/50 split between ages 21–40 and >40 years since 2015 [10]. Previous studies also found that outdoor-associated activities, including occupational exposure and living near a potential vector breeding area, are the key risk factors of leishmaniasis infection in Sri Lanka [11, 12]. These and other studies provide a better understanding of leishmaniasis epidemiology and transmission and pave the way for greater public awareness and disease control. However, further questions remain regarding the spatiotemporal dynamics in leishmaniasis transmission and the driving forces behind it. For example, is the leishmaniasis parasite circulated locally or spread between neighboring areas? This information is important for determining control strategies. Furthermore, since the development of both the sand fly and the parasite inside its gut are affected by climatic conditions [13, 14], how do climatic factors affect leishmaniasis transmission? How can we better utilize this information to predict the future distribution of leishmaniasis in Sri Lanka?

The aim of this study was to further explore spatiotemporal leishmaniasis transmission patterns; to examine the contributions of climatic factors, local carryover of infections, and inter-region transmission to the epidemic situation; and to develop space-time models for the prediction of future clinical case distribution in Sri Lanka. This study can serve as a prototype of an early warning model for the prediction of future epidemics.

Methods

Clinical and climatic data collection

Clinical data collection has been described in a previous study [5]. Briefly, we collected clinically confirmed leishmaniasis case data from the diagnostic and research laboratory at the Faculty of

Medicine, University of Colombo, Sri Lanka. We also obtained data from the Epidemiology Unit of the Ministry of Health, Sri Lanka and through communication with medical health officers in small health administrative units in each district. Case counts of CL at each district covered the entire country from 2001 to 2019; division-level case counts covered the period from 2015 to 2019. We also collected climatic data from meteorological stations; these included maximum, minimum, and mean temperature and precipitation.

Ethics approval for the study was obtained from the ethical review committee, Faculty of Medicine, University of Colombo (Reference number EC-17-062).

Spatial distribution and temporal trend classification

To determine the temporal trend in leishmaniasis transmission, we used the distribution of district-level annual incidence rates and hierarchical clustering to analyze the similarity in incidence rates among different years (spatial similarity) [15]. The purpose of this step was to analyze how transmission had evolved over the past 19 years. We used district-level incidence dynamics and hierarchical clustering to determine the similarity in incidence dynamics among different districts (temporal similarity) [15]. The last step was to measure space-time correlation, i.e., to determine transmission synchrony [16]. This analysis will help to explain the mechanisms behind transmission, e.g., spatial dispersal, locally correlated climatic variables, and/or how vectors might interact with local parasites to produce different patterns of transmission. We used the cross-correlation coefficient to measure region-wide transmission synchrony, and a modified Mantel correlogram to measure local transmission synchrony [16, 17]. We measured correlations only between neighboring districts.

Risk factor analysis

Here we wanted to identify the driving forces behind transmission dynamics in different districts. We tested three types of risk factors: climatic variables, inter-district dispersal, and localized transmission. To assess the risk factors we used the following mixed regression-autoregression model incorporated with a spatial component [18 - 20]:

$$I(D_i, t+1) = \alpha + \beta * I(D_i, t) + \gamma * \sum\nolimits_{ND} I(ND, t) + f(climate, i)$$

$$f(climate, i) = \beta_1 MaxT(D_i, t) + \beta_2 MeanT(D_i, t) + \beta_3 MinT(D_i, t) + \beta_4 Precip(D_i, t)$$

Where $I(D_i, t)$ stands for incidence rate at district *i* in year *t*; *ND* is all neighboring districts of district i; Σ is the sum of all incidence in neighboring districts; *MaxT*, *MeanT*, *MinT* and *Precip* represent the annual average maximum, mean, and minimum temperature and the annual cumulative precipitation, respectively; and α, β, γ, and β_i, i=1–4, are constant coefficients.

The climatic effect was tested to measure if the dynamics of infections were affected by local climatic factors. The neighboring-district effect was tested to determine if the infections in a given district were associated with the dispersal of parasites from neighboring districts. Infections from the previous year were used to test the local infection carryover effect, i.e., the localized transmission trend carried over from the previous year. Risk factor analysis was conducted only in districts where mean annual incidence rate > 5 cases/1,000 population and where climatic observations were available.

We tested each risk factor in an additive fashion; i.e., we first tested the climatic effect, then added the neighboring effect, then added the carryover effect. We used stepwise analysis for variable selection to determine if one factor was more significantly correlated with leishmaniasis incidence dynamics than the other factors.

Space-time prediction

If localized spatiotemporal correlation existed as determined from the previous two steps, and if the climatic effect was not strong, we were able to predict future case distribution based on the carryover and neighboring-district dispersal effects. While this approach may underutilize the climatic information, the model can predict future case numbers without climatic data; we simply cannot use future climatic data (not yet available) to make predictions. Since local transmission and spatial dispersal may occur at a finer scale, we used division-level data to build the following predictive model [20. 21]:

$$I(D_i, t+1) = \alpha + \beta * I(D_i, t) + \gamma * \sum_{ND} I(ND, t)$$

Here, $I(D_i, t)$ is the number of cases in division *i* at time *t*; *ND* represents neighboring divisions of division *i*; and α, β, and γ are constant coefficients.

The data we used to build the model were division-level case numbers from 2015–2018. We used 2019 data for validation. We used the model to predict the case distribution for 2020.

The maps and spatial data were generated using ArcGIS 10.0 (Redlands, CA, USA). Other data analyses were conducted using R 3.6.3.

Results

Leishmaniasis epidemiology: Spatiotemporal dynamics

Case incidences were low from 2001 to 2008, followed by a slow but steady increase in case incidence from 2009 to 2017, until a sudden outbreak occurred in 2018 (Fig 1a). District-level average incidence rate increased about 4-fold from 2017 to 2018. Four districts had average annual incidence rates ≥ 10 cases/1,000 people, and another nine districts had annual incidence rates ≥ 1 case/1,000 people (Fig 1b).

Fig 1. Mean annual incidence rate (cases/1,000 population): a) by year (left), and b) by district (right).

Classification of district-level incidence distribution found that the study period could be classified into three periods based on similar distribution patterns: 2001–2010, 2011–2017, and 2018–2019 (Fig 2). All average incidence rates were < 1 case/1,000 people from 2001 to 2010, between 1 and 5 from 2011 to 2017, and >10 cases/1,000 people after 2017 (Fig 2). The similarity in incidence distributions did not follow chronological order, as indicated in branches of the classification tree (Fig 2). Overall, 2001–2010 could be seen as a low-transmission period, 2011–2017 as a parasite population build-up period, and 2018–2019 as an epidemic period (Fig 1a and 2).

Fig 2. Classification of study period by disease distribution at each district.

Districts could be classified into three groups based on similarity in incidence dynamics (Fig 3). The majority of the districts with low incidence rates were in one group (gray on the map, Fig 3). Four districts with intermediate incidence rates had similar temporal dynamics (yellow on the map). The remaining seven districts (green on the map) were not necessarily classified into one group; however, they had temporal dynamics that differed from the other two groups, and they were also the seven districts with the highest average annual incidence rates (Fig 3). Incidence rates in these seven districts were subjected to further risk factor analysis.

Fig 3. Classification of study areas by disease dynamics at each district.

The heat map in Figure 4 illustrates the distribution of incidence rates over time (Fig 4). Results from the space-time joining classification indicated that the study areas could be roughly classified into three groups: A) continuously low incidence until 2019 (top section); B) relatively high incidence before 2008, followed by a decline and then a resurgence starting in 2012 (middle section); and C) continuously increasing incidence (bottom section) (Fig 4).

Fig 4. Joining classification of study areas and study period by incidence rate at each district.

The global mean correlation among district incidence dynamics was 0.30 (95% CI 0.25–0.35). However, the localized Mantel correlation between neighboring districts was on average 0.58 (95% CI 0.42–0.73), indicating a rather high correlation between neighboring districts.

Risk factor analysis

The seven districts for which we built risk analysis models were all classified into one group based on transmission dynamics (Fig 3), and they all had average annual incidence rates > 5 cases/1,000 people. The climatic variables–only model showed that three out of seven models yielded a correlation coefficient $R^2 > 0.5$, indicating good correlation with climatic factors, while two districts showed no significant correlation between disease dynamics and temperature or precipitation variables (Table 1). Without considering the neighboring-district dispersal and carryover effects, precipitation alone was not significantly correlated ($P < 0.05$) with leishmaniasis transmission dynamics (Table 1).

Table 1. Risk factors of clinical leishmaniasis in different districts.

District	Hambantota	Matara	Kurunegala	Polonnaruwa	Matale	Anuradhapura	Vavuniya
Mean annual incidece rate (cases/1,000 population)	41.817	19.264	6.972	21.779	7.505	23.532	9.550
Climate model							
Adjusted R^2	0.829	N/A	N/A	0.784	0.425	0.711	0.185
ANOVA F-value	30.127			22.812	7.648	23.196	5.079
P-value	<0.0001			<0.0001	0.0047	<0.0001	0.0377
Coefficients							
Minimum temperature	-68.499 ***					-78.453 ***	6.547 *
Mean temperature	64.465 **			83.635 ***	64.183 **		
Maximum temperature	-35.656 *			-37.536 **	-39.912 *	69.320 ***	
Precipitation							
Climate + Neighbor-district model							
Adjusted R^2	0.838	0.808	0.873	0.930	0.832	0.726	0.185
ANOVA F-value	47.675	38.967	62.606	119.738	45.612	24.861	5.079
P-value	<0.0001	<0.0001	<0.0001	<0.0001	<0.0001	<0.0001	0.0377
Coefficients							
Minimum temperature	-35.213 **	22.311 ***					6.547 *
Mean temperature							
Maximum temperature							
Precipitation			-0.005 **	0.004 *	-0.009 ***	0.014 **	
Neighboring-district mean	3.340 ***	1.262 ***	1.815 ***	3.061 ***	1.551 ***	2.444 ***	
Climate + Neighbor-district + Carryover model							
Adjusted R^2	0.915	0.846	0.937	0.930	0.832	0.816	0.316
ANOVA F-value	65.449	33.858	89.557	119.738	45.612	40.796	9.310
P-value	<0.0001	<0.0001	<0.0001	<0.0001	<0.0001	<0.0001	0.0072
Coefficients							
Minimum temperature							
Mean temperature							
Maximum temperature							
Precipitation	0.015 *	-0.009 *	-0.003 *	0.004 *	-0.009 ***		

Note: Stepwise regression analysis was used, insignificant variables were removed at level of 0.05; *, ** and *** represent significant level of <0.05, <0.01 and <0.001, respectively.

When the neighboring-district effect was included, six of the seven models had $R^2 > 0.7$ and the neighboring-district effect appeared in six models ($P < 0.05$), precipitation appeared in four models ($P < 0.05$), and one model still showed very weak correlation (Vavuniya District, $R^2 = 0.18$) between transmission dynamics and risk factors (Table 1).

When climatic, neighboring-district dispersal, and carryover effects were all included, precipitation appeared in five models, neighboring-district effect in six models, and carryover

effect in five models. Temperature did not appear in any of the models tested (Table 1). Once again, models for disease dynamics in Vavuniya District showed weak correlation ($R^2 = 0.32$) with the risk factors investigated; only the carryover effect was selected by the stepwise process, indicating likely locally isolated transmission (Table 1).

Overall, the risk model–predicted disease dynamics matched well with the observed dynamics in six of the seven districts, all but Vavuniya (Fig 5). Leishmaniasis transmission in Vavuniya was likely to be an outlier.

Fig 5. Observed and model-predicted temporal changes in incidence rate in the seven districts with the highest case numbers. Climate, neighbor, and carryover represent climatic, neighboring-district dispersal, and carryover effects, respectively.

Spatial distribution prediction

Using cases from the previous year in the same division and in neighboring divisions, we built a prediction model with $R^2 = 0.823$ and adjusted $R^2 = 0.823$ (ANOVA $F_{2,1321} = 3075.71$, $P < 0.0001$, Table 2). Model-predicted cases matched well with observed cases for all years except 2018, which was an epidemic year (Fig 6 and 7, Supplemental Fig S1). The key mismatch between observed and predicted cases in 2018 was the intensity in the north-central and southwestern coastal areas, where the model underestimated the observations in some high-transmission divisions (Fig 6, Supplemental Fig S1). The model predicted both the expansion of the epidemic area and the increased transmission intensity, as well as the expansion of the southern hotspot from coastal to inland areas (Fig 6). The predicted case number in 2020 is 5,379 (95% CI [4739, 6017]), which is about a 30% increase from 2019 observations (4,064 cases) (Fig 7). If the current trend holds, the predicted case number will be 9,115 (95% CI

[8,157, 10,070]) by year 2022 and close to 20,000 by year 2025 (19,212 cases, 95% CI [17,389, 21031]).

Fig 6. Observed and model-predicted leishmaniasis cases by division and year.

Fig 7. Observed vs. model-predicted total cases from 2015 to 2025.

Supplemental Fig S1. Correlation between observed and predicted cases at each division from 2016 to 2019.

Table 2. Division-level predictive model summary

Variable	Coefficient (95% CI)	t-value	p value
Intercept	0.8102 [0.1921, 1.4284]	2.57	0.0102
Cases from previous year	1.0679 [1.0273, 1.1086]	51.52	< 0.0001
Cases in neighboring divisions from previous year	0.0331 [0.0215, 0.0446]	5.61	< 0.0001

Discussion

Leishmaniasis clinical cases are still on the rise as reported by the Sri Lankan Ministry of Health, in contrast to the substantial decline in case numbers recoded in other South Asian countries including India, Sri Lanka's nearest neighbor [2, 5]. Although leishmaniasis clinical

cases were first reported from Sri Lanka in 1992 [22], the case numbers have remained low and sporadic prior to 2001 [23]. Reported case numbers increased 15-fold from 2001 to 2010, followed by a 10-fold increase from 2010 to 2019, with a clear outbreak in 2017–2018. In addition to localized transmission, inter-area dispersal seems to have played an important role in spreading the parasite, and precipitation is probably the key climatic factor fueling the increasing trends in infections. Our model predicted that cases will continue to rise unless viable interventions are implemented.

Spatiotemporal analysis is a powerful tool, not only for detecting transmission hotspots but also for understanding the mechanisms behind transmission, e.g., how spatial dispersal and locally correlated climatic variables contribute to transmission [11, 15, 16, 24 – 26]. In most previous leishmaniasis epidemiological studies, spatiotemporal analyses were used only for clustering and finding the hotspots [11, 24 -29]. Conventional risk factor analysis is good for finding risk factors, but it lacks predictive power [30, 31]. In previous studies, mixed climate-time-series or pure time-series models have ignored disease spatial spreading, or at least have not utilized the spatial information; usually they have treated each study region independently or used data from only one study region [32 -34]. In this study, we proposed a multi-step approach including not only spatiotemporal clustering but also spatiotemporal risk modeling. Using spatiotemporal classification analysis, we found that the disease dynamics in Sri Lanka from 2001 to 2019 may be divided into three phases: the low-transmission phase from 2001 to 2010, the parasite population build-up phase from 2011 to 2017, and the sudden epidemic starting in 2018. From 2001 to 2010, parasites circulated locally with a few hotspots [5]. Prior to 2017, no more than three districts had relatively high incidence rates (>50 cases/1,000 people-years) each year; however, this number increased to five districts in 2018 and seven in 2019. Spatial correlation analysis indicated that transmission dynamics between neighboring districts were positively correlated, suggesting inter-area spreading. Risk factor analysis in the heavily hit districts suggested that precipitation was likely the key climatic factor driving the increase in cases.

However, what caused the sudden 2018 leishmaniasis outbreak in Sri Lanka is a subject for further investigation. The results from this study provide a better understanding of the epidemiology of leishmaniasis and the factors related to the emergence of the disease. The temporal prediction is useful for epidemic preparedness, and the spatial prediction can help to guide targeted intervention control planning.

Leishmaniasis transmission in Vavuniya District was a special case. Vavuniya and Kilinochchi districts were in the front lines of the 30-year armed conflict in Sri Lanka, which ended in 2009 [35, 36]. With the existence of indigenous circulating leishmaniasis parasite, the transmission of leishmaniasis in Vavuniya District seemed more locally circulated, and might be due to the displacement of locals during the conflict and the postwar return of individuals who had fled, coupled with the influx of armed forces personnel who assisted with resettlement and reconstruction of infrastructure [35, 36]. It was more like a test cage, where parasites circulated freely. Once resettlement was completed, local infections may have been mass tested, clinical cases treated and armed forces personnel redeployed elsewhere [35, 36], which may have influenced the control of parasite transmission. Such factors may explain the failure to reliably predict the transmission dynamics in this district in relation to the climatic variables and neighboring-district dispersal.

A limitation of this study is the spatiotemporal predictive model, which lacked the power to predict the 2018 leishmaniasis outbreak. Since long-term dynamics of climatic variables (for example, next year's climate) are currently unpredictable, any climate-based model lacks predictive power. Climatic factors were not included in the predictive model in this study; however, if climatic factors were indeed the driving force behind the 2018 outbreak, model predictions excluding climatic variables would surely miss the epidemic. Studies in other countries have linked climatic variability with outbreaks of leishmaniasis [37 – 39]. We speculate that climatic variation may be one of the causes of the 2018 outbreak in Sri Lanka; however,

further evidence is required. Another limitation is the short temporal period used for building the predictive model (the model without climatic variables). We collected disease data from some 350 divisions for each year, which provided a good sample size in terms of the goodness-of-fit of the model; still, four years is a short period. Adding data observed from other years might improve model performance, although collecting accurate long-term nationwide division-level clinical case data has proven difficult.

In conclusion, leishmaniasis transmission in Sri Lanka is still spreading and has recently intensified. Spatiotemporal analysis incorporating a neighboring-area dispersal effect provides a new framework for analyzing the spatiotemporal dynamics of the disease, detecting the risk factors that affect the dynamics, and predicting potential future distribution of infections[illegible]uch information on future transmission distribution predictions are useful for disease control programs and policy makers for planning and prioritizing of targeted areas.

Data Availability: The authors confirm that all data underlying the findings are fully available without restriction. All relevant data are within the paper and its Supporting Information files.

Funding: Research reported in this publication was supported by the National Institute of Allergy and Infectious Diseases, National Institutes of Health under award no. U01AI136033 to N.D.K. and G.Z. The authors are solely responsible for the content and it does not necessarily represent the official views of the National Institutes of Health.

Competing Interests: The authors declare that there are no competing interests.

Acknowledgments

We are grateful to the head and staff of the Department of Parasitology, Faculty of Medicine, University of Colombo, and the Director General and staff of the Ministry of Health, especially the medical health officers, other health officials, and epidemiologists, for assistance in this study. We thank Raushan Siraj, Sachee Bhanu, Nishanthan Chandrasekeran, Dasun Dissanayake and Ruksala Ranatunga for assistance with data collection. This study was performed in the Faculty of Medicine of the University of Colombo, Sri Lanka.

Author Contributions

NK and GZ: Conceived, designed the study, interpreted results and wrote the manuscript; SS, SG, HS, NM, NS, RD, PK, DG, NdeS, US: designed the study and collated data. GZ: Analyzed the data.

References

1. WHO. Leishmaniasis. https://www.who.int/health-topics/leishmaniasis#tab=tab_1 (accessed: July 15, 2020)
2. Burza S, Croft SL, Boelaert M. Leishmaniasis. Lancet 2018; **392**:951–70. Rev. https://doi.org/10.1016/S0140-6736(18)31204-2 Epub 2018 Aug 17. PMID: 30126638.
3. WHO, Regional Office for South-East Asia. Regional strategic framework for elimination of kala-azar from the South-East Asia Region (2011-2015). WHO Regional Office for South-East Asia, 2012. https://apps.who.int/iris/handle/10665/205826
4. Rijal S, Sundar S, Mondal D, Das P, Alvar J, Boelaert M. Eliminating visceral leishmaniasis in South Asia: the road ahead. Br Med J 2019;364:k5224. doi: 10.1136/bmj.k5224. PMID: 30670453; PMCID: PMC6340338.
5. Karunaweera ND, Ginige S, Senanayake S, Silva H, Manamperi N, Samaranayake N, et al. Spatial epidemiologic trends and hotspots of Leishmaniasis, Sri Lanka, 2001-2018. Emerg Infect Dis 2020; 26(1):1–10. http://doi.org/10.3201/eid2601.190971 PMID: 31855147; PMCID: PMC6924882.
6. Karunaweera ND, Pratlong F, Siriwardane HV, Ihalamulla RL, Dedet JP. Sri Lankan cutaneous leishmaniasis is caused by *Leishmania donovani* zymodeme MON-37. Trans R Soc Trop Med Hyg 2003; 97:380–1. https://doi.org/10.1016/S0035-9203(03)90061-7 PMID: 15259461.
7. Senanayake SA, Abeyewicreme W, Dotson EM, Karunaweera ND. Characteristics of Phlebotomine sandflies in selected areas of Sri Lanka. Southeast Asian J Trop Med Public Health 2015; 46:994–1004. PMID: 26867357; PMCID: PMC6206496.
8. Gajapathy K, Peiris LBS, Goodacre SL, Silva A, Jude PJ, Surendran SN. Molecular identification of potential leishmaniasis vector species within the *Phlebotomus* (Euphlebotomus) *argentipes* species complex in Sri Lanka. Parasit Vectors 2013; 6:302. https://doi.org/10.1186/1756-3305-6-302 PMID: 24499561; PMCID: PMC3853795.

9. Rajapaksa US, Ihalamulla RL, Udagedera C, Karunaweera ND. Cutaneous leishmaniasis in southern Sri Lanka. Trans R Soc Trop Med Hyg 2007;101(8):799-803. doi:10.1016/j.trstmh.2006.05.013
10. Siriwardana Y, Zhou G, Deepachandi B, Akarawita J, Wickremarathne C, Warnasuriya W, et al. Trends in recently emerged *Leishmania donovani* induced cutaneous leishmaniasis, Sri Lanka, for the first 13 years. BioMed Res Int 2019; 2019:e4093603. https://doi.org/10.1155/2019/4093603 PMID: 31111052; PubMed Central PMCID: PMC6487155.
11. Kariyawasam KK, Edirisuriya CS, Senerath U, Hensmen D, Siriwardana HV, Karunaweera ND. Characterisation of cutaneous leishmaniasis in Matara district, southern Sri Lanka: evidence for case clustering. Pathog Glob Health 2015;109(7):336-343. doi:10.1179/2047773215Y.0000000032
12. Ranasinghe S, Wickremasinghe R, Munasinghe A, Hulangamuwa S, Sivanantharajah S, Seneviratne K, et al. Cross-sectional study to assess risk factors for leishmaniasis in an endemic region in Sri Lanka. Am J Trop Med Hyg 2013; 89:742–9. https://doi.org/10.4269/ajtmh.12-0640 PMID: 23918217; PubMed Central PMCID: PMC3795106.
13. Dostálová A, Volf P. Leishmania development in sand flies: parasite-vector interactions overview. Parasit Vectors 2012; 5:276. https://doi.org/10.1186/1756-3305-5-276 PMID: 23206339; PMCID: PMC3533922.
14. Poché DM, Grant WE, Wang HH. Visceral Leishmaniasis on the Indian Subcontinent: modelling the dynamic relationship between vector control schemes and vector life cycles. PLoS Negl Trop Dis 2016; 10(8):e0004868. https://doi.org/10.1371/journal.pntd.0004868 PMID: 27537774; PMCID: PMC4990243.

15. Witten IH, Frank E, Hall MA, Pal CJ. Data Mining: Practical Machine Learning Tools and Techniques. Morgan Kaufmann Series in Data Management Systems. 4th ed. Morgan Kaufmann, San Diego, 2016.
16. Bjørnstad ON, Ims RA, Lambin X. Spatial population dynamics: analyzing patterns and processes of population synchrony. Trend Ecol Evol 1999; 14:427–33. https://doi.org/10.1016/s0169-5347(99)01677-8 PMID: 10511718.
17. Koenig WD. Spatial autocorrelation of ecological phenomena. Trends Ecol Evol 1999; 14:22–26. https://doi.org/10.1016/s0169-5347(98)01533-x PMID: 10234243.
18. Anselin L, Gallo JL, Jayet H. Spatial Panel Econometrics. In: Mátyás L, Sevestre P (eds) The Econometrics of Panel Data. Advanced Studies in Theoretical and Applied Econometrics, vol 46. Springer, Berlin, Heidelberg, 2008. pp 625-660. https://doi.org/10.1007/978-3-540-75892-1_19
19. Elith J, Leathwick JR. Species distribution models: ecological explanation and prediction across space and time. Annu Rev Ecol Evol Syst 2009; 40:677–97. https://doi.org/10.1111/gcb.12548 PMID: 24526603.
20. Kyriakidis PC, Journel AG. Geostatistical space–time models: a review. Math Geol 1999; 31:651–84. https://doi.org/10.1023/A:1007528426688
21. Cliff AD, Ord JK. Space-time modelling with an application to regional forecasting. Trans Institute British Geographers 1975; 64:119–28.
22. Athukorale DN, Seneviratne JK, Ihalamulla RL, Premaratne UN. Locally acquired cutaneous leishmaniasis in Sri Lanka. J Trop Med Hyg 1992; 95:432–3. PMID: 1460704.
23. Karunaweera ND. *Leishmania donovani* causing cutaneous leishmaniasis in Sri Lanka: a wolf in sheep's clothing? Trends Parasitol 2009; 25:458–63. https://doi.org/10.1016/j.pt.2009.07.002 PMID: 19734098.

24. Bhunia GS, Kesari S, Chatterjee N, Kumar V, Das P. Spatial and temporal variation and hotspot detection of kala-azar disease in Vaishali district (Bihar), India. BMC Infect Dis 2013; 13:64. https://doi.org/10.1186/1471-2334-13-64 PMID: 23375077; PMC3577657.
25. Rodríguez E-M, Díaz F, Pérez M-V. Spatio-temporal clustering of American Cutaneous Leishmaniasis in a rural municipality of Venezuela. Epidemics 2013; 5(1):11–9. https://doi.org/10.1016/j.epidem.2012.10.002 PMID: 23438427.
26. Melchior LAK, Brilhante AF, Chiaravalloti-Neto F. Spatial and temporal distribution of American cutaneous leishmaniasis in Acre state, Brazil. Infect Dis Poverty 2017; 6(1):99. https://doi.org/10.1186/s40249-017-0311-5 PMID: 28587683; PMCID: PMC5461694.
27. Dewan A, Abdullah AYM, Shogib MRI, Karim R, Rahman MM. Exploring spatial and temporal patterns of visceral leishmaniasis in endemic areas of Bangladesh. Trop Med Health 2017;45:29. doi: 10.1186/s41182-017-0069-2.
28. Mandal R, Kesari S, Kumar V, Das P. Trends in spatio-temporal dynamics of visceral leishmaniasis cases in a highly-endemic focus of Bihar, India: an investigation based on GIS tools. Parasit Vectors 2018;11(1):220. doi:10.1186/s13071-018-2707-x
29. Zheng C, Fu J, Li Z, Lin G, Jiang D, Zhou XN. Spatiotemporal variation and hot spot detection of visceral leishmaniasis disease in Kashi Prefecture, China. Int J Environ Res Public Health 2018; 15(12):2784. https://doi.org/10.3390/ijerph15122784 PMID: 30544811; PMCID: PMC6313707.
30. Golpayegani AA, Moslem AR, Akhavan AA, Zeydabadi A, Mahvi AH, Allah-Abadi A. Modeling of environmental factors affecting the prevalence of zoonotic and anthroponotic Cutaneous, and zoonotic Visceral Leishmaniasis in foci of Iran: a remote sensing and GIS based study. J Arthropod Borne Dis 2018; 12(1):41–66. PMID: 30018993; PMCID: PMC6046107.
31. Ding F, Wang Q, Fu J, Chen S, Hao M, Ma T, et al. Risk factors and predicted distribution of visceral leishmaniasis in the Xinjiang Uygur Autonomous Region, China, 2005-2015. Parasit

Vectors 2019; 12(1):528. https://doi.org/10.1186/s13071-019-3778-z PMID: 31703720; PMCID: PMC6839266.

32. Chaves LF, Calzada JE, Valderrama A, Saldaña A. Cutaneous Leishmaniasis and sand fly fluctuations are associated with Niño in Panamá. PLoS Negl Trop Dis 2014; 8(10): e3210. https://doi.org/10.1371/journal.pntd.0003210 PMID: 25275503; PMCID: PMC4183471.

33. Ferreira de Souza RA, Andreoli RV, Toshie Kayano M, Lima Carvalho A. American cutaneous leishmaniasis cases in the metropolitan region of Manaus, Brazil: association with climate variables over time. Geospat Health 2015; 10(1):314. https://doi.org/10.4081/gh.2015.314 PMID: 26054516.

34. Galgamuwa LS, Dharmaratne SD, Iddawela D. Leishmaniasis in Sri Lanka: spatial distribution and seasonal variations from 2009 to 2016. Parasit Vectors 2018; 11(1):60. https://doi.org/10.1186/s13071-018-2647-5 PMID: 29370864; PubMed Central PMCID: PMC5785883.

35. Gunathilaka N, Semege S, Pathirana N, Manamperi N, Udayanga L, Wijesinghe H, et al. Prevalence of cutaneous leishmaniasis infection and clinico-epidemiological patterns among military personnel in Mullaitivu and Kilinochchi districts of the Northern Province, early war-torn areas in Sri Lanka. Parasit Vectors 2020;13(1):263. https://doi.org/10.1186/s13071-020-04137-8 PMID: 32430014; PMCID: PMC7236442.

36. Semage SN, Pathirana KPN, Agampodi SB. Cutaneous leishmaniasis in Mullaitivu, Sri Lanka: a missing endemic district in the leishmaniasis surveillance system. Int J Infect Dis 2014; 25:53–5. https://doi.org/10.1016/j.ijid.2014.03.1382 PMID: 24858902.

37. Chaves LF, Pascual M. Climate cycles and forecasts of Cutaneous Leishmaniasis, a nonstationary vector-borne disease. PLoS Med 2006; 3(8):e295. https://doi.org/10.1371/journal.pmed.0030295 PMID: 16903778; PubMed Central PMCID: PMC1539092.

38. Li Y, Zheng C. Associations between meteorological factors and Visceral Leishmaniasis outbreaks in Jiashi County, Xinjiang Uygur Autonomous Region, China, 2005–2015. Int J Environ Res Public Health 2019; 16:1775. https://doi.org/10.3390/ijerph16101775 PMID: 31137482; PMCID: PMC6571646.

39. Nikonahad A, Khorshidi A, Ghaffari HR, Aval HE, Miri M, Amarloei A, et al. A time series analysis of environmental and metrological factors impact on cutaneous leishmaniasis incidence in an endemic area of Dehloran, Iran. Environ Sci Pollut Res 2017; 24:14117–23. https://doi.org/10.1007/s11356-017-8962-0 PMID: 28417326.

Legends to Figures

Figure 1. Mean annual incidence rate (cases/1,000 population): a) by year (left), and b) by district (right).

Figure 2. Classification of study period by disease distribution at each district.

Figure 3. Classification of study areas by disease dynamics at each district.

Figure 4. Joining classification of study areas and study period by incidence rate at each district.

Figure 5. Observed and model-predicted temporal changes in incidence rate in the seven districts with the highest case numbers. Climate, neighbor, and carryover represent climatic, neighboring-district dispersal, and carryover effects, respectively.

Figure 6. Observed and model-predicted leishmaniasis cases by division and year.

Figure 7. Observed vs. model-predicted total cases from 2015 to 2025.

Supplemental Figure S1. Correlation between observed and predicted cases at each division from 2016 to 2019.

Figure 1

Click here to access/download;Figure;Fig 1.pptx

Figure 1. Nationwide mean annual incidence rate (cases/1,000 people/year) from 2001 to 2019 (a) and the distribution of average incidence rate in each district over the study period (b)

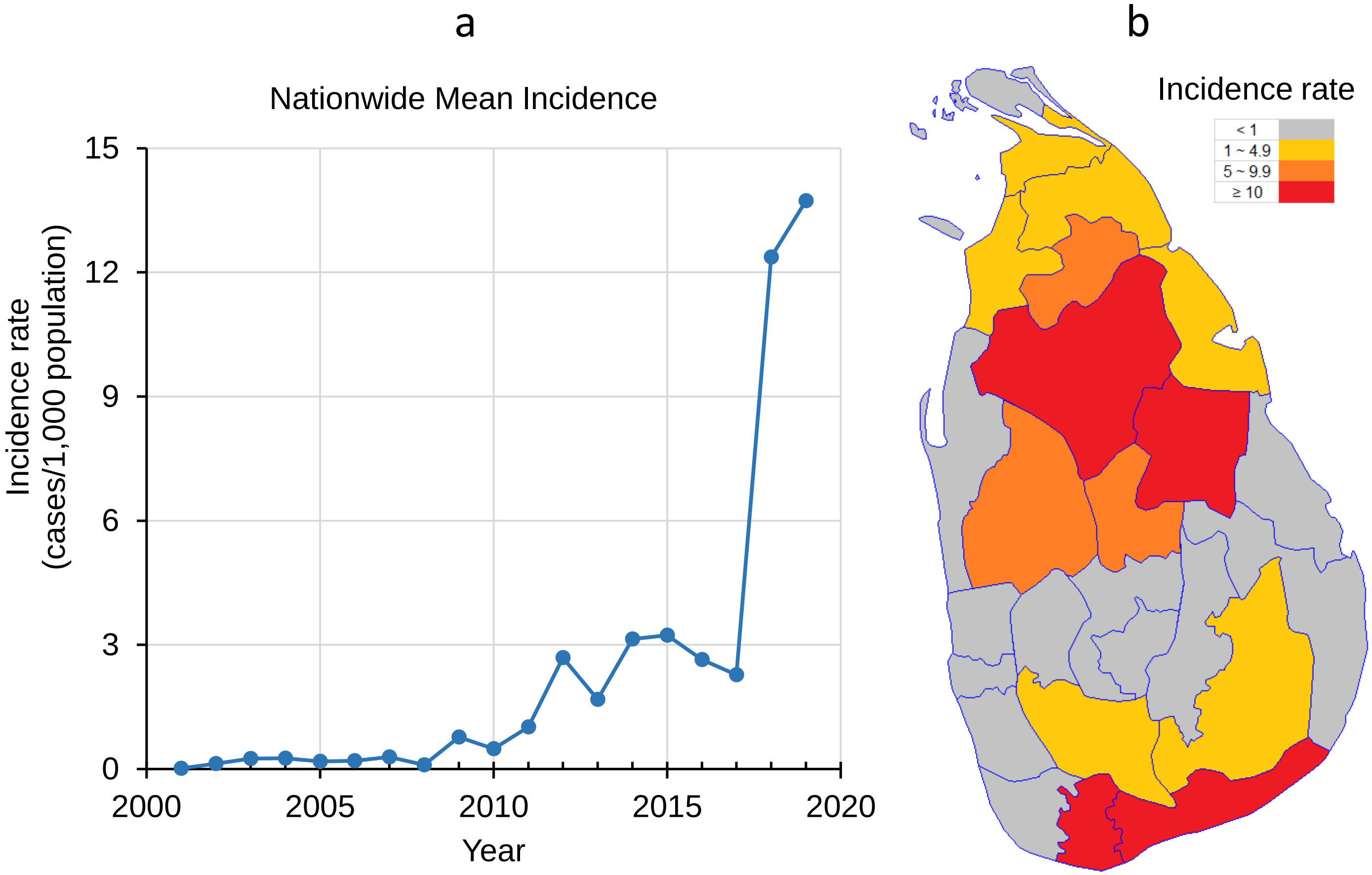

Figure 2 Click here to access/download;Figure;Fig 2.pptx

Fig 2. Classification of study period by disease distribution at each district

Year	IR
2001	0.02
2002	0.13
2005	0.18
2006	0.19
2007	0.29
2008	0.10
2003	0.25
2004	0.26
2009	0.77
2010	0.48
2011	1.02
2015	3.23
2016	2.64
2012	2.69
2014	3.14
2013	1.68
2017	2.28
2018	12.37
2019	13.73

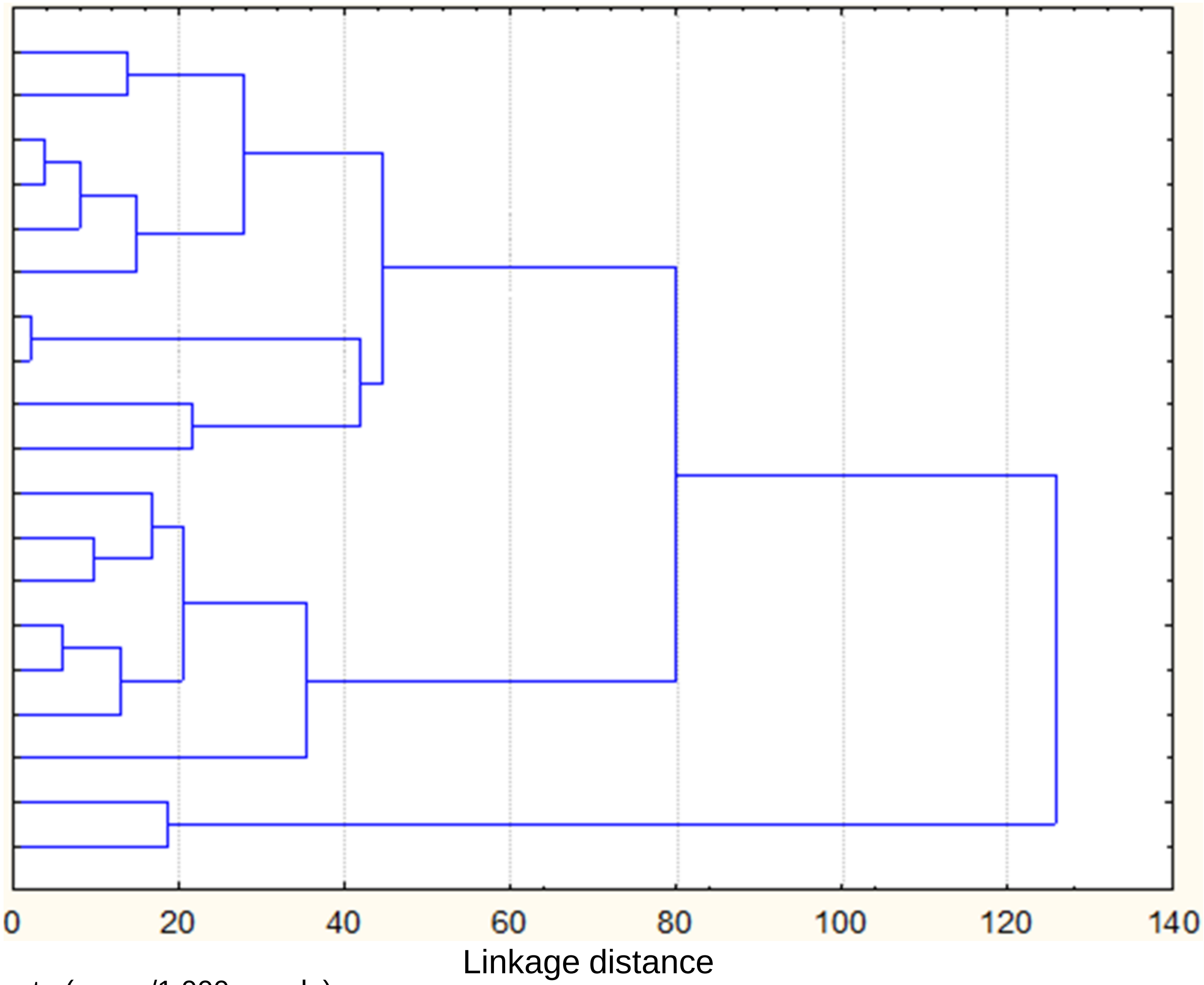

* IR: Incidence rate (cases/1,000 people)

Figure 3 Click here to access/download;Figure;Fig 3.pptx

Fig 3. Classification of study areas by disease dynamics at each district. The colour on the map match the ones showing in the District/IR table

District	IR
Ampara	0.45
Puttalam	0.42
Badulla	0.46
Batticaloa	0.07
NuwaraEliya	0.05
Galle	0.27
Kalutara	0.16
Colombo	0.26
Jaffna	0.47
Trincomalee	2.32
Mannar	1.69
Kandy	0.66
Gampaha	0.65
Kegalle	0.67
Monaragala	2.86
Kilinochchi	2.50
Ratnapura	2.82
Mullaitivu	4.66
Vavuniya	9.16
Kurunegala	6.97
Matale	7.50
Matara	19.26
Anuradhapura	25.46
Polonnaruwa	21.78
Hambantota	41.82

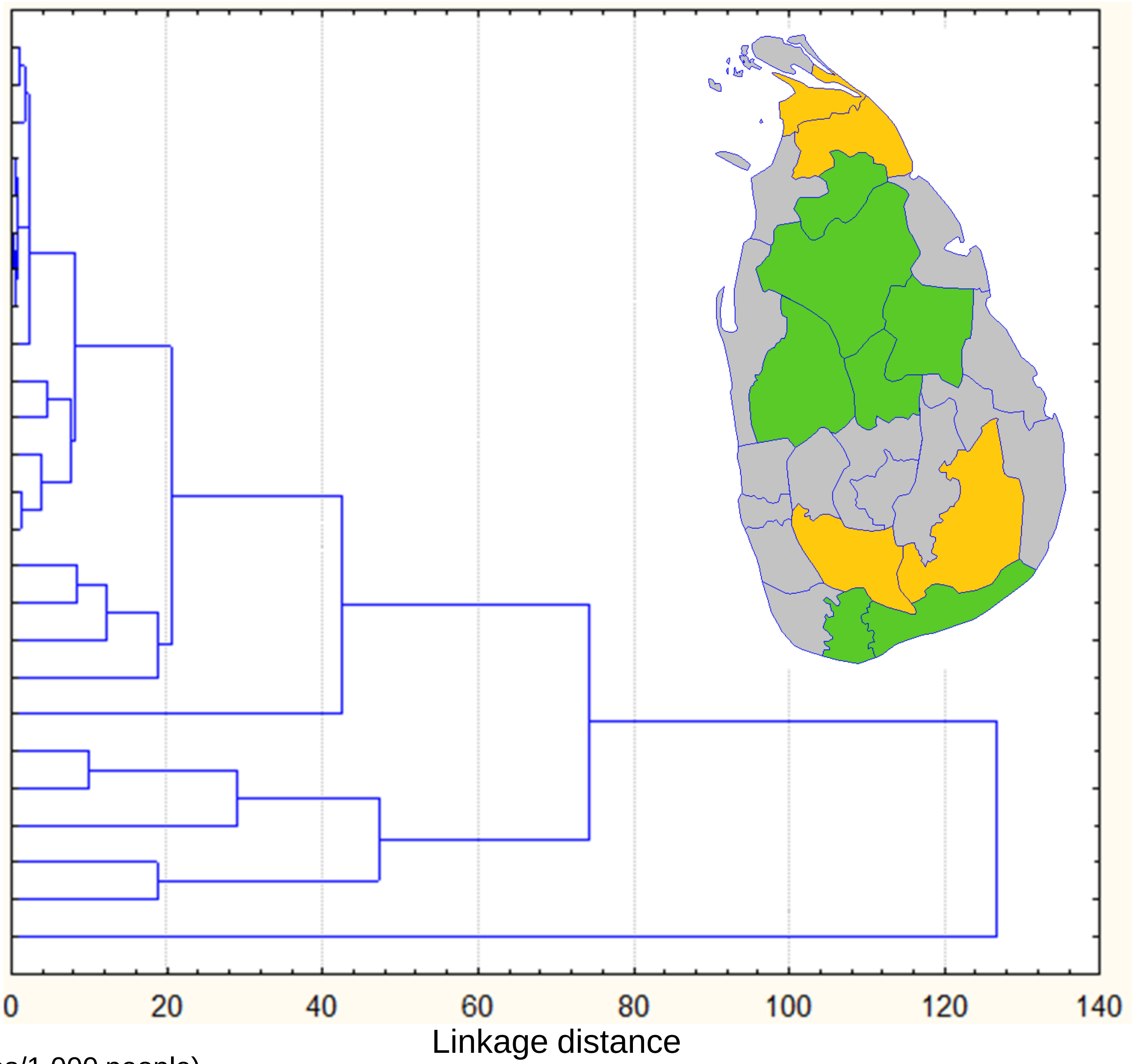

IR: Incidence rate (cases/1,000 people)

Figure 4

Click here to access/download;Figure;Fig 4.pptx

Fig 4. Joining classification of study areas and disease dynamics

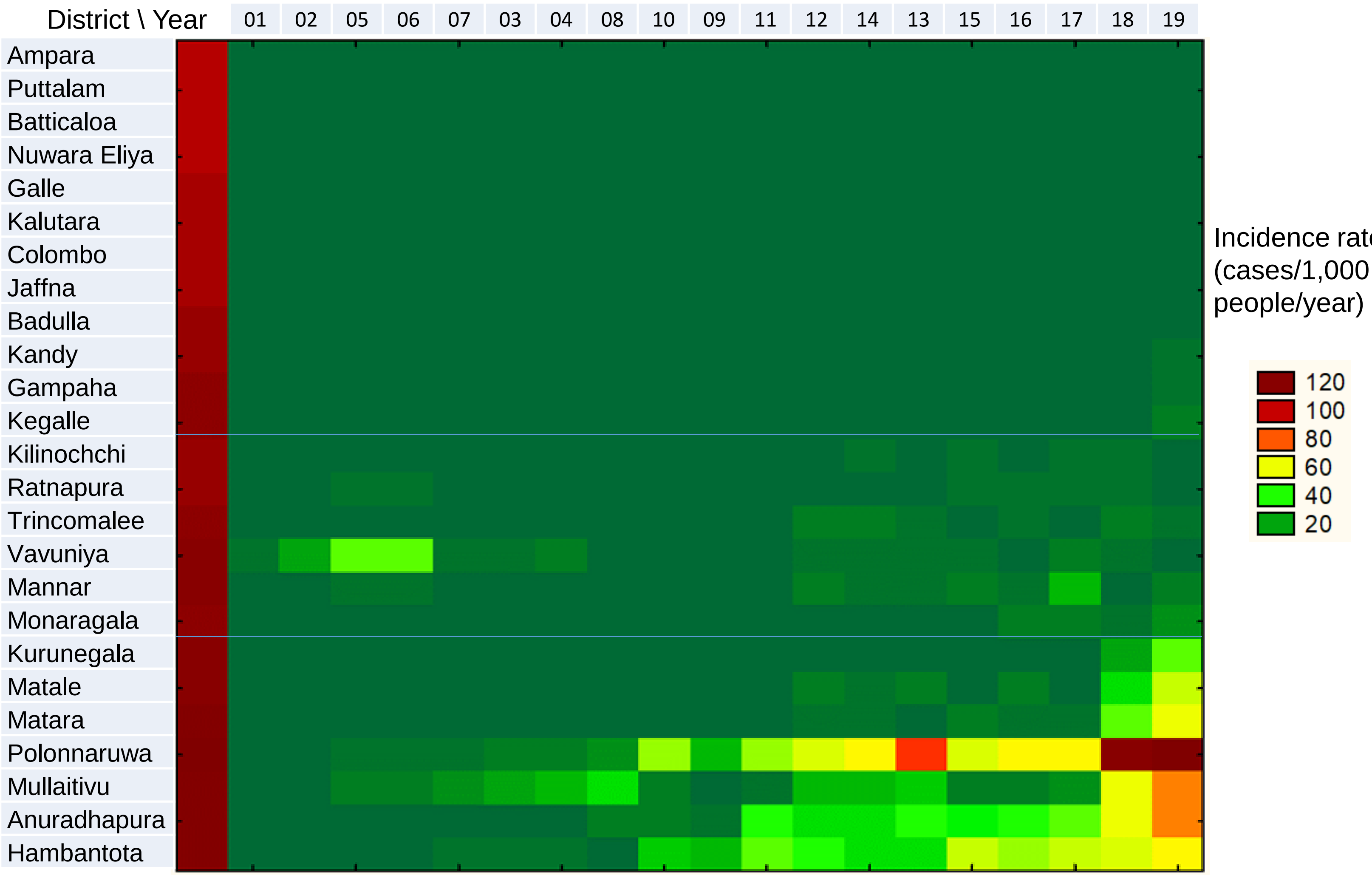

Figure 5

Click here to access/download;Figure;Fig 5.pptx

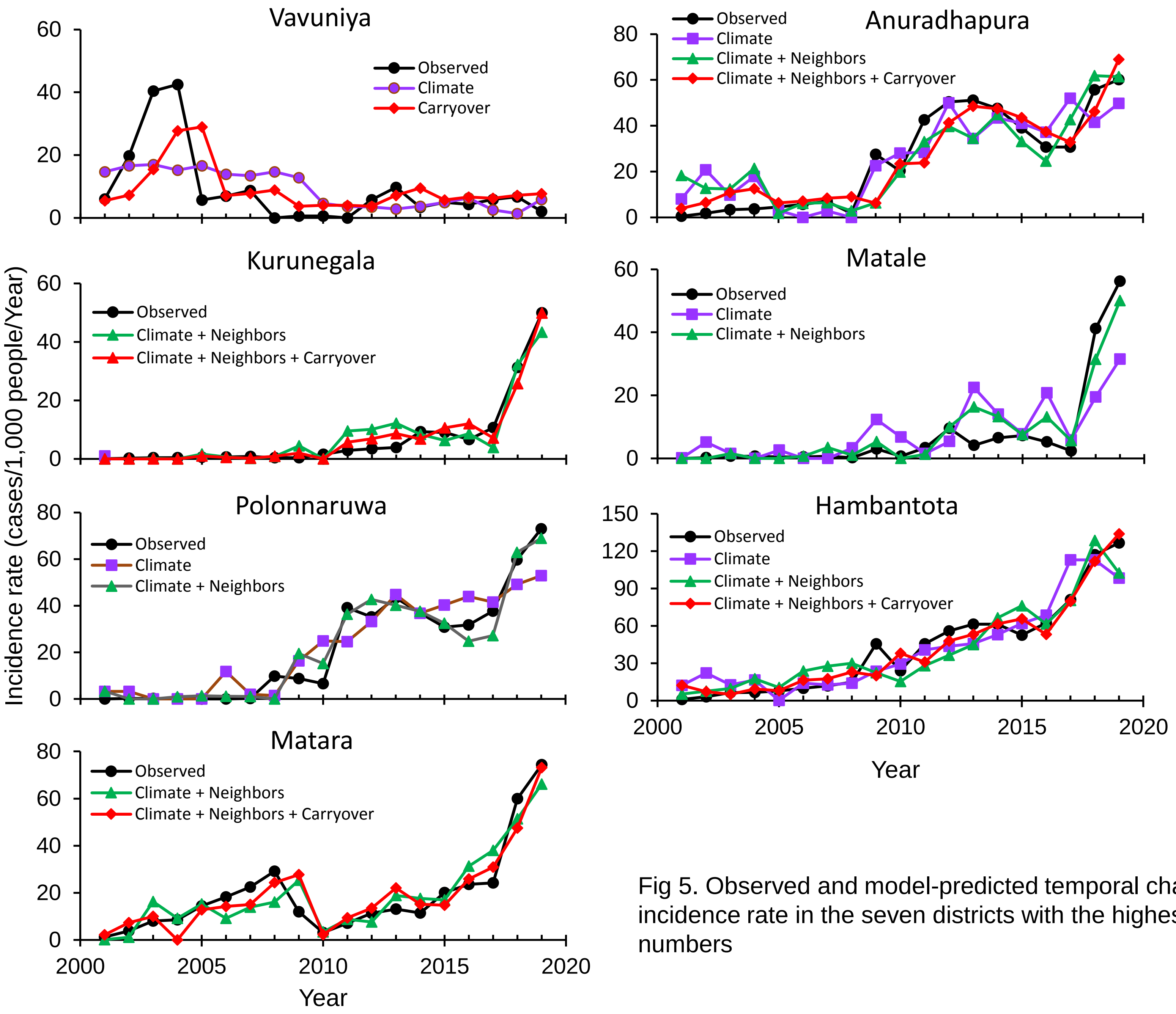

Fig 5. Observed and model-predicted temporal changes in incidence rate in the seven districts with the highest case numbers

Figure 6

Click here to access/download;Figure;Fig 6.pptx

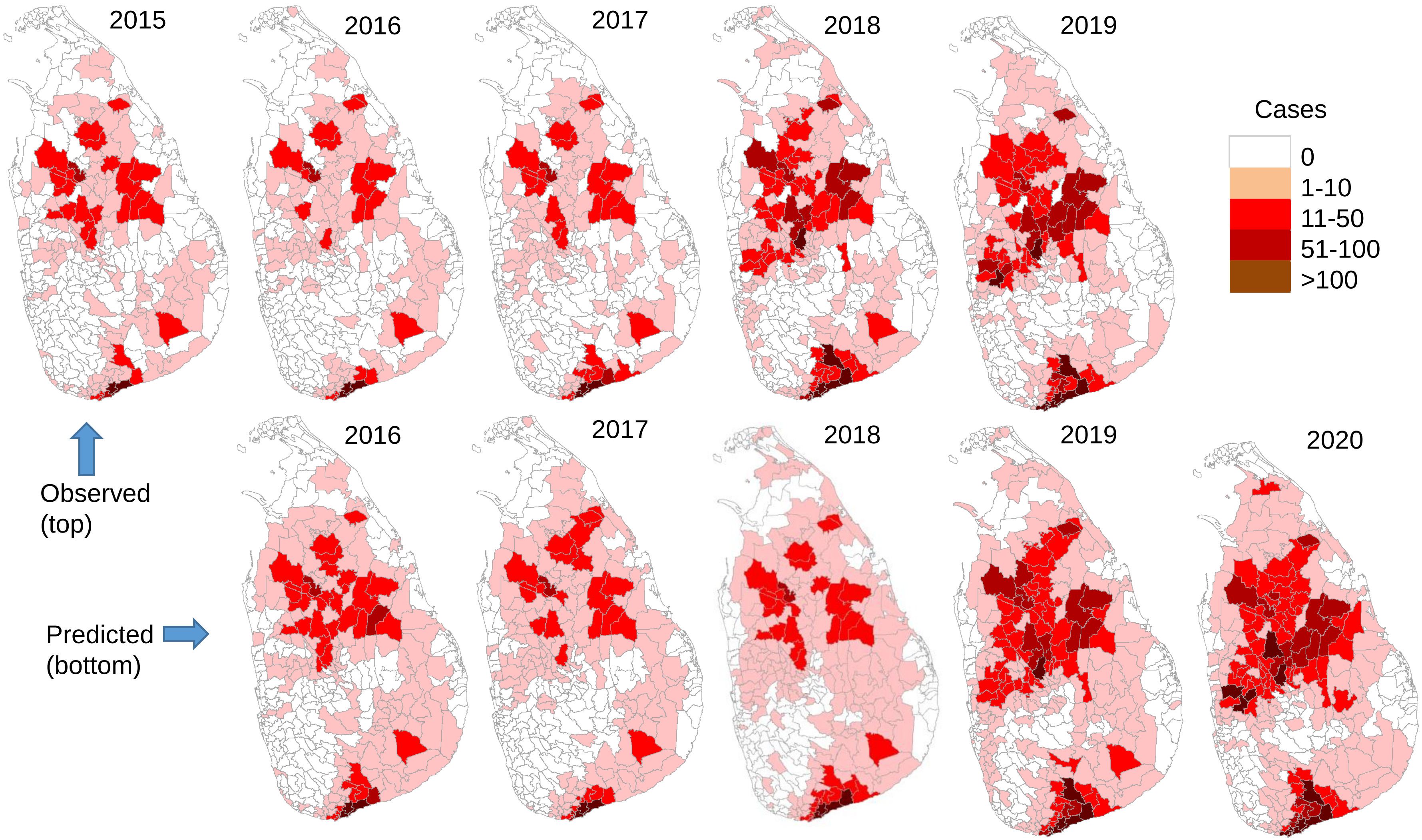

Fig 6. Observed and model-predicted leishmaniasis cases by division and year

Figure 7

Click here to access/download;Figure;Fig 7.pptx

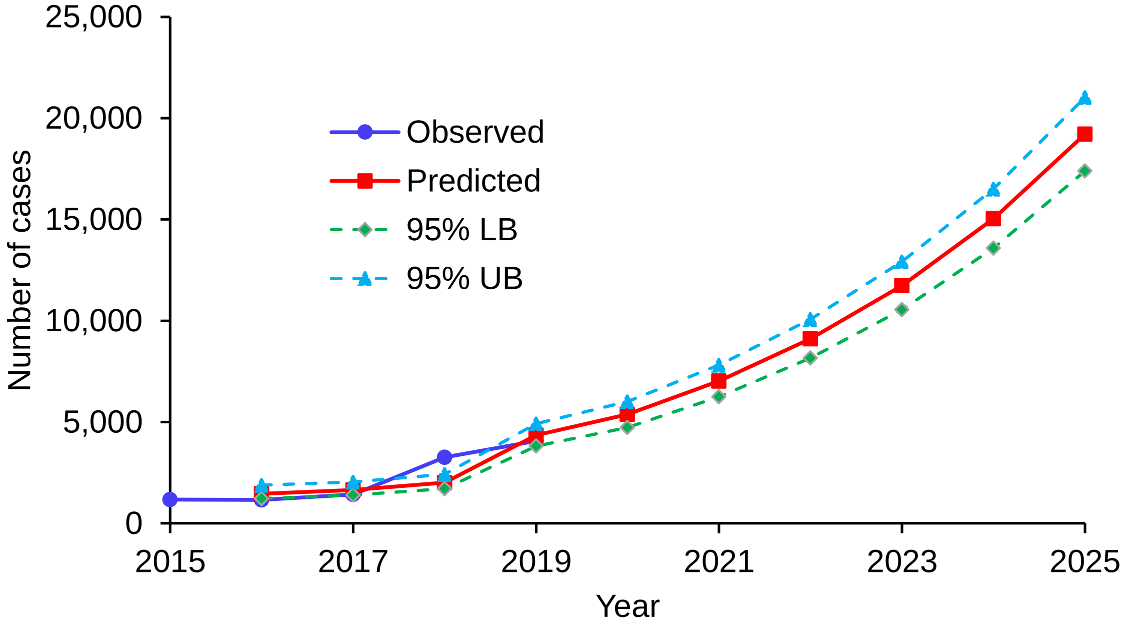

Fig 7. Observed (2015-2019) vs. model-predicted (2016-2025) total cases. 95% LB/UB represent the lower and upper boundaries of the 95% confidence interval of the predictions

Click here to access/download;Figure;Suppl Fig S1.pptx

Supplement Fig S1. Correlation between observed and predicted cases at each division from 2016 to 2019

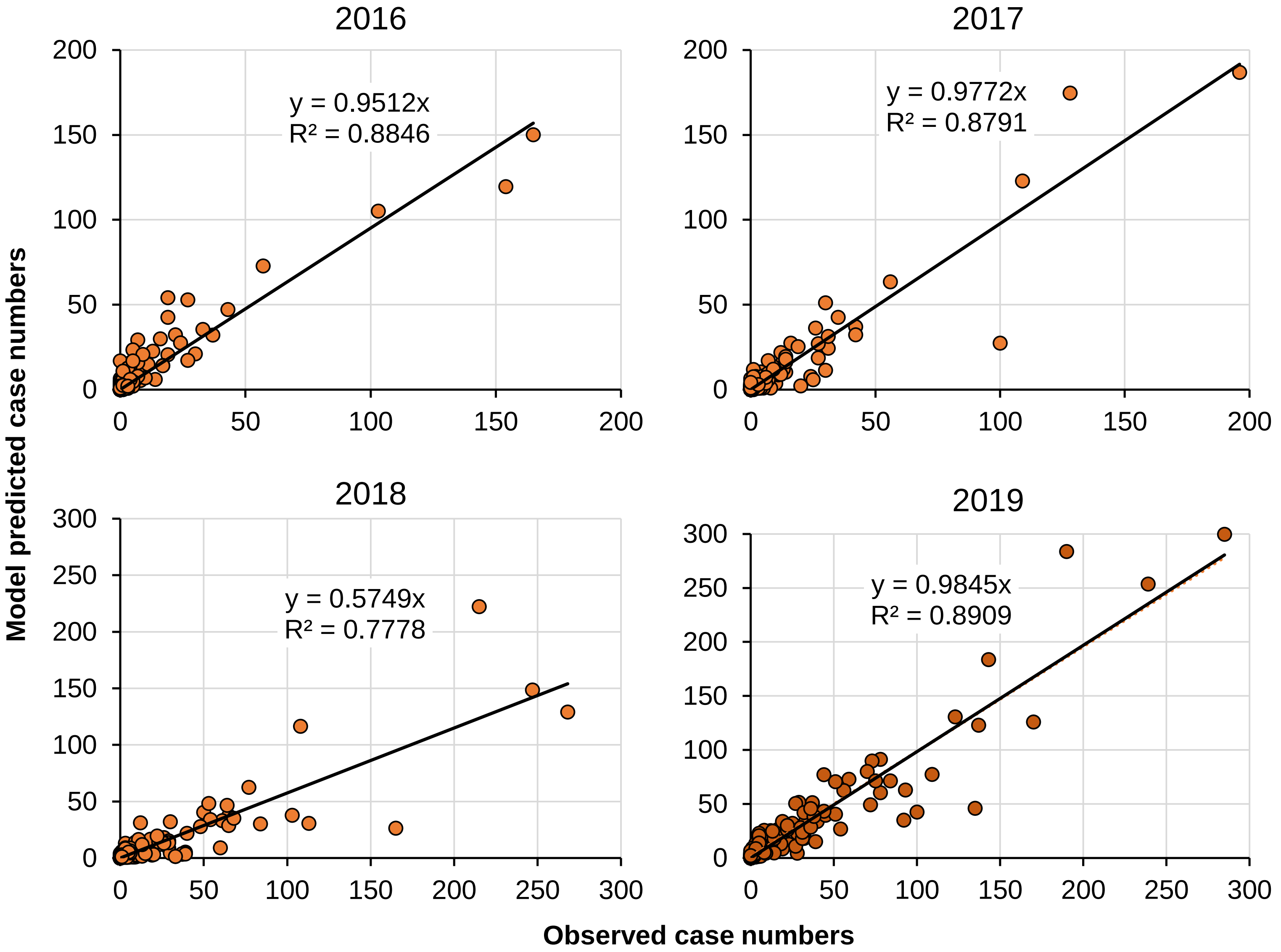

Supplement: Attachment — Submitted filename: PNTD-D-20-02228_reviewer-1.pdf [file pntd.0009346.s002.pdf]
