# Supplementary material for: Spatiotemporal distribution of cutaneous leishmaniasis in Sri Lanka and future case burden estimates
Source: PLoS Negl Trop Dis. 2021 Apr 23;15(4):e0009346. doi: 10.1371/journal.pntd.0009346 (PMC8099137; doi:10.1371/journal.pntd.0009346)
Supplement: Attachment — Submitted filename: Rewiever Comments.pdf [file pntd.0009346.s003.pdf]

Dear editor and authors, I had read with interest the manuscript "Spatiotemporal transmission of cutaneous leishmaniasis in Sri Lanka and future case burden estimates". The idea of the study is really interesting since this research used the spatiotemporal analysis and disease risk factors to understand transmission mechanisms of the disease and to predict future disease distribution. Overall, the manuscript is interesting but will need major improvement before being accepted for publication. Furthermore, I recommend some minors revisions as following.

Best regards

Major issues:

- It would make the article stronger if the authors clarified how this study will serve as useful for health decision-makers. It needs to be clear what the tangible result will lead to after your paper is read by a diverse audience.
- It is not clear why Sri Lanka must be studied, and the level of significance it will have for other regions of the world or studies that require a similar methodological approach. There need to be major revisions to this section (literature review) and clarify why the Sri Lanka case study discussed in this paper is necessary and how this particular study actually contributes to the scientific field.
- Previous studies analyzed the spatial and temporal behavior of different types of leishmaniasis around the world. Please clarify key findings of the present work comparing previously published literature.
- The authors should mention further limitations of the research in the discussion section.

Minor issues:

- Lines 66 and 131: In this study, what type of cutaneous leishmaniasis has been studied? Zoonotic Cutaneous Leishmaniasis (ZCL) and Anthroponotic Cutaneous Leishmaniasis (ACL)? Please clear it.
- Line 92: Please use a comma before using "and".
- Line 93: Please use a comma before using "and".
- Line 113-114: This sentence needs a proper citation, which explains leishmaniasis epidemiology. Please include the following suggestion at the end of the sentence.

- Spatiotemporal variability of Zoonotic Cutaneous Leishmaniasis based on sociodemographic heterogeneity. The case of Northeastern Iran, 2011-2016. (M Tabasi, AA Alesheikh).

- The introduction section needs more citation, which includes works done on spatiotemporal modeling of leishmaniasis. Please add the following suggestions in the this section:

  - A spatio-temporal agent-based approach for modeling the spread of zoonotic cutaneous leishmaniasis in northeast Iran. (M Tabasi, AA Alesheikh, A Sofizadeh, B Saeidian, B Pradhan, A AlAmri).

  - Development of an Agent Based Model for Simulation of the Spatiotemporal Spread of Leishmaniasis in GIS (Case Study: Marveh Tappeh). (M Tabasi, AA Alesheikh).

- Lines 147-148: To better identify spatial or space-time disease clusters, you can use space-time scan statistics, which have been used in the following research:

  - Spatiotemporal variability of Zoonotic Cutaneous Leishmaniasis based on sociodemographic heterogeneity. The case of Northeastern Iran, 2011-2016. (M Tabasi, AA Alesheikh).
- The meaning of the phrase “infection carryover effect" is unclear. Please explain it better.
- Some of the sentences are repeated in different parts of the manuscript. Authors should avoid writing the same sentence if they are necessary to repeat again.
- Lines 84 to 85 are textually copied from the following article (Chaara D, Ravel C, Bañuls AL, Haouas N, Lami P, Talignani L, El Baidouri F, Jaouadi K, Harrat Z, Dedet JP, Babba H. Evolutionary history of Leishmania killicki (synonymous Leishmania tropica) and taxonomic implications. Parasites & vectors. 2015 Dec;8(1):1-9). Use other related sentences to prevent plagiarism.
- Grammar mistakes can be seen throughout the manuscript at times; it distracts from the content. Scientific, proofreading, and editing by a native speaker are necessary.
