# Supplementary material for: Spatiotemporal distribution of cutaneous leishmaniasis in Sri Lanka and future case burden estimates
Source: PLoS Negl Trop Dis. 2021 Apr 23;15(4):e0009346. doi: 10.1371/journal.pntd.0009346 (PMC8099137; doi:10.1371/journal.pntd.0009346)
Supplement: Attachment — Submitted filename: Responses to reviewers comments.docx [file pntd.0009346.s004.docx]

**Author responses to reviewers’ comments as listed under each section of the manuscript:**

*Methods:*

Reviewer #5:

*Query (Q):* Please mention from where did you obtain meteorological data?

*Response (R):* This information is given under materials and methods section in the manuscript “climatic data was obtained from 20 meteorological stations” with more details added to improve clarity (see lines 185-189).

*Q:* How many clinically confirmed cases you included for this study?
*R:* The total number of confirmed cases (19361) is now added at the beginning of the “Results” section (line 265) and in the abstract (line 41).

*Q:* What are the tests you applied to analyze risk factors?
*R:* The statistical methods used for risk factor analyses is now detailed out in the materials and methods section. See lines 237-242.

*Results*
Reviewer #5:

*Q:* Fig 1 (b) incidence rate - which year?

*R:* In the Figure caption, we described as “average incidence rate in each district over the study period”.

*Q:* Add p values for each clinical factors in every districts
*R:* p-values have been provided for each district in Table 1.

**Queries as listed under Overall Comments:**

**Reviewer #1**

*Query:* The data of figure 5 is not explained well in the text. I recommend mentioning about it more detail in the text, or move this figure into a supplemental figure.
*Response:* We agree. Thanks. Now more details have been added in the figure 5 caption to address this deficiency.

**Reviewer #2**

Major issues:

1. It would make the article stronger if the authors clarified how this study will serve as useful for health decision-makers. It needs to be clear what the tangible result will lead to after your paper is read by a diverse audience.

*Response:* We have discussed the potential implications of our results in the “Discussion” (lines 352-360; 368-383) and “Conclusions” (420-425) sections.

2. It is not clear why Sri Lanka must be studied, and the level of significance it will have for other regions of the world or studies that require a similar methodological approach. There need to be major revisions to this section (literature review) and clarify why the Sri Lanka case study discussed in this paper is necessary and how this particular study actually contributes to the scientific field.

*Response:*

We agree with the reviewer, on the importance of the contributions of the current study to the scientific field. Furthermore, authors also believe that all studies are important regardless of study area. While some studies may focus on local diseases, the wider impact of findings and conclusions drawn may have either regional or global implications, such as the current study described in the article, though all studies don’t necessarily need to have direct worldwide impact.

We have now reviewed relevant studies conducted in Sri Lanka, described the need for the study of leishmaniasis in Sri Lanka in the “Introduction” section stating the importance of this study and how the methodological approach in this study will contribute to the scientific field (second & third paragraphs; lines 96 to 148).

3. Previous studies analyzed the spatial and temporal behavior of different types of leishmaniasis around the world. Please clarify key findings of the present work comparing previously published literature.

*Response:* We have discussed the difference between the present study and previous published works in the introduction section lines 122 to 148.

4. The authors should mention further limitations of the research in the discussion section.

*Response:* We have discussed limitations of this study in Discussion section lines 396 to 418.

Minor issues:

5. Lines 66 and 131: In this study, what type of cutaneous leishmaniasis has been studied? Zoonotic Cutaneous Leishmaniasis (ZCL) and Anthroponotic Cutaneous Leishmaniasis (ACL)? Please clear it.

*Response:* We have emphasized this study focused on anthroponotic cutaneous leishmaniasis. The details are given in Methods section, line 173.

6. Line 92: Please use a comma before using “and”.

*Response:* Change has been made in the text.

7. Line 93: Please use a comma before using “and”.

*Response:* We have changed that as the reviewer suggested.

8. Line 113-114: This sentence needs a proper citation, which explains leishmaniasis epidemiology. Please include the following suggestion at the end of the sentence. Spatiotemporal variability of Zoonotic Cutaneous Leishmaniasis based on sociodemographic heterogeneity. The case of Northeastern Iran, 2011-2016. (M Tabasi, AA Alesheikh).

*Response:* We agree. The stated study by Tabasi & Alesheikh’s is cited (Ref. 13] and results have been discussed (see Introduction section, Lines 122-148).

9. The introduction section needs more citation, which includes works done on spatiotemporal modeling of leishmaniasis. Please add the following suggestions in the this section:
   a. A spatio-temporal agent-based approach for modeling the spread of zoonotic cutaneous leishmaniasis in northeast Iran. (M Tabasi, AA Alesheikh, A Sofizadeh, B Saeidian, B Pradhan, A AlAmri).
   b. Development of an Agent Based Model for Simulation of the Spatiotemporal Spread of Leishmaniasis in GIS (Case Study: Marveh Tappeh). (M Tabasi, AA Alesheikh).

*Response:* Thanks for this suggestion. We have added a section to discuss what have been done with these models and what questions remain with these previous studies, i.e., why this study is needed. See lines 122 to 148.

10. Lines 147-148: To better identify spatial or space-time disease clusters, you can use space-time scan statistics, which have been used in the following research: Spatiotemporal variability of Zoonotic Cutaneous Leishmaniasis based on sociodemographic heterogeneity. The case of Northeastern Iran, 2011-2016. (M Tabasi, AA Alesheikh).

*Response:* Our previous study have already shown the clustering of leishmaniasis cases in Sri Lanka [5], this study focused on: A) space-time synchrony of transmission, B) driving factors for the spatial-temporal dynamics, and C) predicting future distribution and trends of leishmaniasis in Sri Lanka. Authors feel that the studies the reviewer listed here did not have such power to predict the future distributions and trends of leishmaniasis transmission, which is now explained in the Introduction section as detailed above.

11. The meaning of the phrase “infection carryover effect" is unclear. Please explain it better.

*Response:* We added some more explanation. See lines 229 to 234.

12. Some of the sentences are repeated in different parts of the manuscript. Authors should avoid writing the same sentence if they are necessary to repeat again.

*Response:* We do apologize for this and have now avoided such repetitions.

13. Lines 84 to 85 are textually copied from the following article (Chaara D, Ravel C, Bañuls AL, Haouas N, Lami P, Talignani L, El Baidouri F, Jaouadi K, Harrat Z, Dedet JP, Babba H. Evolutionary history of Leishmania killicki (synonymous Leishmania tropica) and taxonomic implications. Parasites & vectors. 2015 Dec;8(1):1-9). Use other related sentences to prevent plagiarism.

*Response:* We do apologize for this. The sentence has now been rephrased.

14. Grammar mistakes can be seen throughout the manuscript at times; it distracts from the content. Scientific, proofreading, and editing by a native speaker are necessary.

*Response:* Language corrections have been done in the latest version with the help of a native English speaker.

**Reviewer #3:**

The paper ‘Spatiotemporal transmission of cutaneous leishmaniasis in Sri Lanka and future case burden estimates’ covers a very relevant and urgent topic in the Sri Lank context.

Below you can find recommendations to further improve this investigation:

1. L99 – Please provide a detailed information about this upsurge in leishmaniasis cases. This is critical to justify the need for the present study.

*Response:* We have added details to describe the leishmaniasis situation in Sri Lanka. See lines 100 to 112.

2. L128-138 – The sections about the study area and data sources is very incomplete. The following information needs to be included:
   2a. Description of the territorial units of analysis, more precisely population size (mean and SD or median and IQR)

*Response:* We have describe the territorial units used in the study, i.e., districts and division. We have also included a short description about the population data used. As we have directly used district/division total population, no mean or SD or median or IQR is necessary since these numbers were used to calculate the incidence rates directly. See lines 158 to 170.

2b. Description of the information systems that collect and provide data on leishmaniosis cases (flow of information from diagnosis until register in the database; coverage; existence of changes in data quality through time, etc.)

*Response:* This study is to analyze government health facility reported cases, i.e., aggregated reported data, not a study to follow individual patient or how the data system works. We believe the information, such as the flow of information from diagnosis until register, is out of the scope of this study; especially the data quality changes is out of our control.

2c. Description of the climatic data, more precisely the data source, temporal resolution and number of meteorological stations in the climate network from Sri Lanka. If data sources are prone to any type of information and selection bias, that needs to be discussed in the limitations section of the manuscript.

*Response:* We describe the climatic data with more details as the reviewer suggested. We have discussed the potential limitations of using climatic data. See lines 172 to 189.

3. L148 – Please clarify the concept of transmission synchrony. The readers may not know this term.

*Response:* We have added a short section in Methods section to describe the spatial synchrony and the primary mechanisms. See lines 202 to 207.

4. L190 – Please indicate the R packages used for the analysis.

*Response:* We added the four packages used, car, lme4, MASS and synchrony in Methods section. See lines 259 to 261.

5. L180 – The authors do not use climatic variable to make the space-time predictions. However, the authors found that climatic factors played a role in the infections. Could it be possible to make predictions based on meteorological scenarios (high, low, medium precipitation year; high, low, medium temperature year)? I believe it would be an interesting add-on to the present or future research.

*Response:* Yes, we agree. It’s definitely interesting to try. However, since our space-time predictions are based on division-level disease data, and division level climatic data is not available, therefore, we could not make any such predictions, which is unfortunate and is described in the text (lines 413 to 418).

6. Figure 1 – To make the figure more informative I recommend numbering the districts and add the names as a legend of the figure.

*Response:* We have added the names and incidence rate in each district as part of the legend.

**Reviewer #4:
(numbered based on the order appeared in the text)**

1. Title: Using “Spatiotemporal clustering” or “Spatiotemporal distribution” instead.

*Response:* We have now changed the title to “Spatiotemporal distribution …”

2. Should be based on MeSH system.

*Response:* We have checked all keywords with MeSH on Demand, the keywords available are all correct, some keywords are not available on MeSH on Demand.

3. Abstract. “Background” should be revised and rewrite based on title of manuscript.

*Response:* We have revised the “Background” section of the ‘Abstract’ as suggested by the reviewer. See lines 28 to 31.

4. Abstract. “Methods: effect of climatic, neighboring-district”, just climatic?

*Response:* In this study, we included “the effects of climatic, neighboring-district dispersal, and infection carryover variables on leishmaniasis dynamics and distribution” as described in the “Methods” section. See lines 36 to 39.

5. Abstract. “Conclusion”, this part is the results, you should write the conclusion.

*Response:* We have revised the section with conclusion. See lines 53 to 57.

6. Line 85. Some female species.

*Response:* We have added “female” into the sentence. Line 85 to 86.

7. Line 87. Need reference for “the most disabling form of the disease”, 92 countries change to 102 countries.

*Response:* Reference included [1] and number of countries (92) has been changed to 102 according to the new WHO report (lines 88 to 91).

8. Lines 91-92. Need reference.

*Response:* References have been added [1,2]. Lines 91 to 95.

9. Lines 113-114. References needed.

*Response:* References are added [11-13]. Lines 120 to 124.

10. Lines 118-121. These sentences in the introduction section should not be questions.

*Response:* The questions given there remain unanswered so far. Therefore, the authors would like to retain those questions, which we believe would be important to the reader since the current paper describe our attempts in addressing those questions.

11. Methods: Need study area?

*Response:* We added a short paragraph to describe the study area. Lines 158 to 170.

12. Line 195. “until a sudden outbreak occurred”, what a sudden outbreak?

*Response:* We have stated that “case numbers increased from 1,508 cases in 2017 to 3,271 in 2018.” See lines 265 to 268.

13. Line 310. “for understanding the mechanisms behind transmission”, how?

*Response:* We replaced “mechanism” with “driving factors” to clarify that. See lines 362 to 364.

14. Line 362. “predicting potential future distribution of infections”, in detail please.

*Response:* This is conclusion, details have been shown in the results section, for example, predicting the distribution of incidence in 2020 (Figure 6) and case numbers from 2020 to 2025 (Figure 7). Therefore, authors feel sufficient information has been provided in the results section that has helped to draw the stated conclusions.

**Reviewer #5:**

1. Don't start sentences as "we.................. Please write in a scientific way.

*Response:* PLOS NTD allows for first person form such as “we” as the starting words. However, we have made all possible attempts to avoid using the first person form at the beginning of sentences.

2. Please give your suggestions more about how a high correlation between neighboring districts and incidence rate of leishmaniasis in endemic regions.

*Response:* We added the range of R-square values in the results to show the significance of the correlations. Lines 310 to 314.
